# TAPPING NATURAL NETWORKS TO ADDRESS DEMENTIA IN A LATINO COMMUNITY

**DOI:** 10.1093/geroni/igz038.2165

**Published:** 2019-11-08

**Authors:** Caroline Gelman, Nancy Giunta

**Affiliations:** 1 Silberman School of Social Work, New York, United States; 2 Silberman School of Social Work, Hunter College, New York, New York, United States

## Abstract

The need for health education regarding Alzheimer’s disease and related dementias (ADRD), specifically designed for Latinos, has been well-documented. Many Latino older adults and their families delay seeking formal help for ADRD symptoms due to lack of information and access to culturally sensitive services. This paper presents preliminary findings of community-based participatory research to develop El Barrio SHARE, a culturally-tailored intervention tapping natural helpers (NHs) to address a need identified by community members in East Harlem, NY. It trains people who often interact with elders in the course of their work (e.g., hairdressers, bodega clerks), and are well-positioned to observe and detect ADRD-related problems and potentially link elders to relevant services. Findings from a process evaluation developing the 10-module NH training suggest that (a) participants seek information that debunks myths and stigma surrounding ADRD, and (b) the need for culturally-tailored, participant-centered interventions in marginalized communities is critical.

